# STARKAP Protocol: preliminary assessment of safety and tolerability of dostarlimab in combination antiretroviral therapy (cART)-refractory HIV associated Kaposi’s Sarcoma

**DOI:** 10.1186/s12885-025-14326-2

**Published:** 2025-07-01

**Authors:** Claudia A. M. Fulgenzi, Alessia Dalla Pria, Alberto Giovanni Leone, Maria Martinez, Elena Ferrer Martinez Del Peral, Maria Eleanor Flores, Mark Bower, David J. Pinato

**Affiliations:** 1 Department of Surgery and Cancer, Imperial College London, Hammersmith Hospital, Du Cane Road, London, W120 NN UK; 2 National Centre for HIV Oncology, Chelsea Westminster Hospital, London, UK; 3 Fondazione IRCCS Istituto Nazionale Dei Tumori, Milan, Italy; 4 Research and Development, Chelsea and Westminster Hospital, Fulham Road, Chelsea, London, SW10 9 NH UK; 5 Department of Translational Medicine, Università del Piemonte Orientale UPO, Via Solaroli 17, Novara, NO 28100 Italy; 6 Section of Virology, Department of Infectious disease, Imperial College London, London, United Kingdom

**Keywords:** Kaposi's sarcoma, Immunotherapy, HIV

## Abstract

**Background:**

Kaposi sarcoma (KS) is a mesenchymal malignancy induced by human herpes virus-8 (HHV-8). Whilst combined anti-retroviral therapy (cART) has substantially reduced the incidence of KS in people living with HIV (PLWH), KS remains the commonest neoplasia in this population. In up to 15–20% of cases, KS fails to respond to cART. T-cells immune exhaustion is thought to play a central role in mediating KS development and progression in PLWH receiving cART. The high reliance of PD-1 in shaping the cART-refractory phenotype poses a strong rationale for the use of PD-1 blocking antibodies against KS.

**Aims:**

StarKap is a phase Ib, open label, single arm study aiming at evaluating the safety, tolerability of dostarlimab- an anti PD-1 antibody- in cART-refractory KS. Secondary endpoint is the evaluation of overall response rates by AIDS Clinical Trial Group (ACTG) criteria complemented by RECIST v1.1 in patients with visceral disease.

**Methods:**

The trial will enrol 20 patients in total. After completion of the screening procedures, participants will receive the first dose of dostarlimab within 28 days. Dostarlimab will be administered at the dose of 500 mg evry 3 weeks for the first 5 cycles, and at 1000 mg every 6 weeks thereafter. Treatment will continue until disease progression, unacceptable toxicity, or completion of 1 year of treatment. The first 6 patients will be enrolled in a safety-lead-in phase, if no dose-limiting toxicities (DLTs) will occur within the 21 days in the first 6 participants, trial will continue. Patients will be monitored for the emergence of adverse events until 90 days after the last dose of treatment. Tumour response will be assessed as objective response rate (ORR) at cycle 4, cycle 8 and end of treatment. Tissue, bloods, and stool samples will be collected at baseline, on treatment and at end of treatment to satisfy the related translation plan.

## Background and rationale

### Background

HIV associated Kaposi’s sarcoma (KS) is a mesenchymal malignancy associated with Human-Herpesvirus 8 (HHV-8) infection, occurring in people living with HIV (PLWH) [1]. The grade of immunosuppression and the CD4 count represent the main risk factor for developing KS in PLWH. Therefore, the incidence of KS has drastically dropped since the widespread availability of combined anti-retroviral therapy (cART). However, PLWH remain a higher risk of KS compared to the general population even in the case of well-controlled viremia, and about 15% of HIV associated KS develop or progress despite HIV control maintained for at least 3 months, and preserved CD4 count (> 100 cells/µL) [1]. These cases are defined as cART-refractory KS, and palliative cytotoxic chemotherapy (e.g., liposomal doxorubicin, paclitaxel, bleomycin) remains the only avaiable therapeutic option. Despite initial response occurring in up to 60% of patients, most of the cases recur with consequent long-term need for chemotherapy leading to cumulative toxicities and impaired quality of life [2]. Immuno-modulatory agents like lenalidomide and pomalidomide are emerging as promising therapy for KS [3], however their use is not currently licensed in all countries and toxicity of these therapies is significant. We have therefore designed a phase Ib trial testing dostarlimab (anti-PD1) in patients affected by cART-refractory KS.

### Rationale

Immune exhaustion is emerging as a leading mechanism responsible for the development and progression of cART-refractory KS. Studies specifically conducted in patients affected by cART-refractory KS have reported higher grade of CD8 + T cells mitochondrial dysfunction [4] and features of immune senescence in PLWH and KS compared to PLWH without KS [5]. HHV-8 infection is thought to be pivotal in leading to immune dysfunction: in fact, both pre-clinical and clinical studies have reported HHV-8 to directly induce immune exhaustion by leading to PD-1/PD-L1 pathway overactivation in both tumour [6] and immune cells [7]. Furthermore, translational experiments from our group have demonstrated the presence of immune infiltrate to be prominent in KS lesions, and to be associated with PD-L1 over-expression in cART- refractory KS tumour samples [8]. These results suggest the PD-1/PD-L1 pathway to be exploited by KS tumour cells to evade CD8 + T cells attack within the tumour micro-environment. Reverting this mechanism by direct PD-1 inhibition might be able to restore adequate CD8 + T cells function against both HHV-8 and KS.

Immune-checkpoint inhibitors (ICIs) have been tested both as monotherapy (pembrolizumab) [9] and in combination (ipilimumab plus nivolumab) [10] in KS occurring in people without HIV, with promising results. Pembrolizumab has been recently tested in a single arm phase II study, with an objective response rate (ORR) of about 70% according to the AIDS clinical trial group (ACTG) criteria [9]. Among the 17 patients treated with pembrolizumab, 6 had not received any prior chemotherapy. Similarly, a phase 2 study testing the combination of the anti CTLA-4 antibody ipilimumab plus the anti PD-1 nivolumab has been tested in KS patients without HIV, after progression on at least 1 line of prior systemic therapy, showing an ORR of 87% according to RECIST without new safety concerns [10]. In both trials, responses were independent from the type and the number of previous treatment lines.

So far, ICIs in HIV-associated KS have been tested in only one dedicated prospective trial. This is a phase Ib trial conducted in the US, which recently reported on the safety and efficacy of pembrolizumab (anti-PD1) in HIV-associated KS. The trial included patients with undetectable viral load and a CD4 count of at least 50 cells/µL. Among the 32 patients enrolled, 31% ad at least one immune-related AE (irAE) with 25% required systemic steroids. The ORR according to the ACTG criteria was 62.1%. The safety and the efficacy profiles did not differ according to the CD4 count [11]. Furthermore, no HIV-related complications, nor CD4 count alterations were reported during the study. This has been also confirmed in the other available studies testing ICI in PLWH for different oncological indications [12–14]. A cohort (N = 15) of patients affected by KS was also included in the AIDS Malignancy Consortium 095 Study, a multicentre phase I study, which tested nivolumab (anti-PD1) at 3 mg/kg every 2 weeks in PLWH with undetectable HIV-RNA and CD4 count of at least 100 cells/µL affected by solid tumours or KS. The incidence of adverse events of any causes in the whole cohort was 80%, and 25% of the participants experienced grade 3 or higher adverse events. Fatigue, nausea, diarrhea, rash, and lymphopenia were the most common AEs at least possibly related to nivolumab occurring in more than 10% of the subjects. All of which of grade 1 or 2. No significant changes in the HIV-RNA or CD4 count occurred in the study. The ORR per ACTG criteria in the KS subgroup was 40% [12].

No safety nor efficacy data are currently available for dostarlimab in PLWH. According to the latest product’s brochure, with a data cut-off in April 2024, overall, 2087 participants without HIV have been treated in clinical trials with dostarlimab either alone or in combination therapy. The safety profile of the monotherapy from the GARNET [15] phase III trial, enrolling 494 patients affected by advanced endometrial cancer progressing to platinum-based chemotherapy, reported an incidence of all causes adverse events in any patients, with nausea (53.9%), alopecia (53%) and fatigue (51.9%) being the most common. The incidence of grade 3 or higher adverse events was 70.5% in the dostarlimab arm. The most common immune-related (AE) were hypothyroidism (11.2%), skin rash (6.6%), arthralgia (5.8%), and alanine aminotransferase increase (5.8%) [15].

These results, along with the emerging data supporting the safety of immune checkpoint inhibitors in PLWH, prompt the specific investigation of this approach in cART-refractory KS.

We have therefore designed an investigator lead study to assess the safety and activity of dostarlimab- an anti PD1 antibody- in a cohort of 20 patients affected by cART- refractory KS.

## Clinical trial registration

NCT05646082 (ClinicalTrials.gov). Registration date: 02–12–2022. Star date: 26–05–2023.

## Methods

### Trial design

The StarKap study is a single centre open label phase Ib trial assessing the safety, tolerability and preliminary efficacy of dostarlimab in patients affected by cART-refractory KS. The trial is sponsored by Imperial College London and is currently open to recruitment at Chelsea & Westminster Hospital, in London, UK.

A phase Ib, single-arm, open label study describing safety and tolerability of dostarlimab is necessary to answer the question of feasibility of anti-PD-1 immunotherapy in PLWH and KS. No dose-escalation was deemed necessary in this population due to the non-linear relationship between exposure and immune-mediated toxicity and the lack for dose-limiting toxicities observed in dose escalation studies of dostarlimab [15, 16].

The study will enrol 20 adult patients aged (aged ≥ 16 years) affected by HIV-related KS, fulfilling the criteria of cART-refractory KS. To this end, patients will be required to have KS that progressed or remained stable after at least 3 continous months of cART, with HIV-RNA < 200 copies/ml and CD4 count of at least 100 cell/µL at screening. Upon completion of screening procedures and confirmation of eligibility, patients will start treatment with dostarlimab within 28 days from screening. Dostarlimab will be administered intra-venously according to summary of product characteristics, at the dose of 500 mg every 3 weeks for the first 4 cycles and at 1000 mg every 6 weeks for the following cycles. Dostarlimab will be continued until completion of 1 year, loss of clinical benefit, inacceptable toxicities or withdrawal of consent, whichever comes first.

A safety lead-in phase will initially require a total of 6 patients, who will be observed for the emergence of dose-limiting-toxicities (DLTs) over a 21-days window from dosing. Following discussion within the trial steering committee it was opted to maintain a standard 21-days safety window for the first 6 subjects with a particular focus on the assessment of events of immune-reconstitution as well as qualitative and quantitative changes in parameters reflective of HIV control. The decision to enrol 6 subjects for the initial safety lead-in phase is in line with standard clinical trial design practice for early-phase trials. This number is generally considered sufficient to detect any early DLTs [17].

Once all the 6 patients have completed the 21-day safety window observation, trial steering committee (TSC) will review the AE profile and, in the absence of DLTs advise on continuation of recruitment to the subsequent expansion phase leading to enrolment of 20 patients in total. If ≥ 1 DLTs will occur within the first 21 days in the patients enrolled in the safety-lead-in phase, the trial will be prematurely interrupted. During the TSC review, the enrolment of new patients will be paused. In the absence of AEs preventing to do so, the treatment of the first 6 patients will continue as planned, without waiting for the TSC review. The definition of DLTs is reported in Table 1. No dose-reductions are allowed. In general, dostarlimab must be withheld for drug-related toxicities of grade 3, but may be resumed upon recovery to Grade ≤ 1; dostarlimab will be permanently discontinued for any drug-related AE of grade 4. Treatment delays for up to 21 days are permitted in the case of AEs. Any AEs requiring a treatment interruption > 21 days will prompt treatment interruption.

**Table 1:** Definition of dose limiting toxicities according to CTCAE v.5

**Dose limiting toxicities**	
Any treatment-related Grade ≥3 nonhematologic clinical toxicity excluding: − Nausea and vomiting resolving to ≤ grade 1 within 48 hours − Grade 3 diarrhea with duration < 48 hours − Grade 3 fatigue with duration < 7 days − Infusion related reaction − Any treatment-related non hematology toxicity specifically defined as: − ≥Grade 2 uveitis, eye pain, or blurred vision that does not resolve with topical therapy within 2 weeks − ≥Grade 2 immune related endocrine toxicity that requires hormone replacement (except Grade 2 thyroiditis or thyroid dysfunction) − ≥Grade 2 colitis or diarrhea that persists for ≥ 7 days despite adequate steroid therapy − Any toxicity that results in a treatment delay of ≥7 days − Any treatment-related Grade ≥3 non-hematologic laboratory abnormality if: − Medical intervention is required to treat the patient, or − The abnormality leads to hospitalization, or − The abnormality persists for ≥7 days − Any treatment-related hematologic toxicity specifically defined as: − Grade 4 thrombocytopenia for ≥7 days, or grade 3 or 4 associated with bleeding or requiring platelet transfusion − Grade 4 neutropenia for ≥7 days, or grade 3 or 4 associated with infection or febrile neutropenia − Grade 4 anaemia, or grade 3 anaemia requiring blood transfusion	

Tumoral skin biopsies will be collected at baseline, during treatment (cycle 4), and at the end of treatment to complete the associated translational plan. Stools, bloods and urine samples will be collected at baseline, at cycle 4, at cycle 8 and end of treatment.

Participants will be followed at 30 and 90 days after removal from protocol therapy or until death, whichever occurs first. Overall survival will be followed for 2 years following the last dose of protocol therapy.

**Fig. 1:**
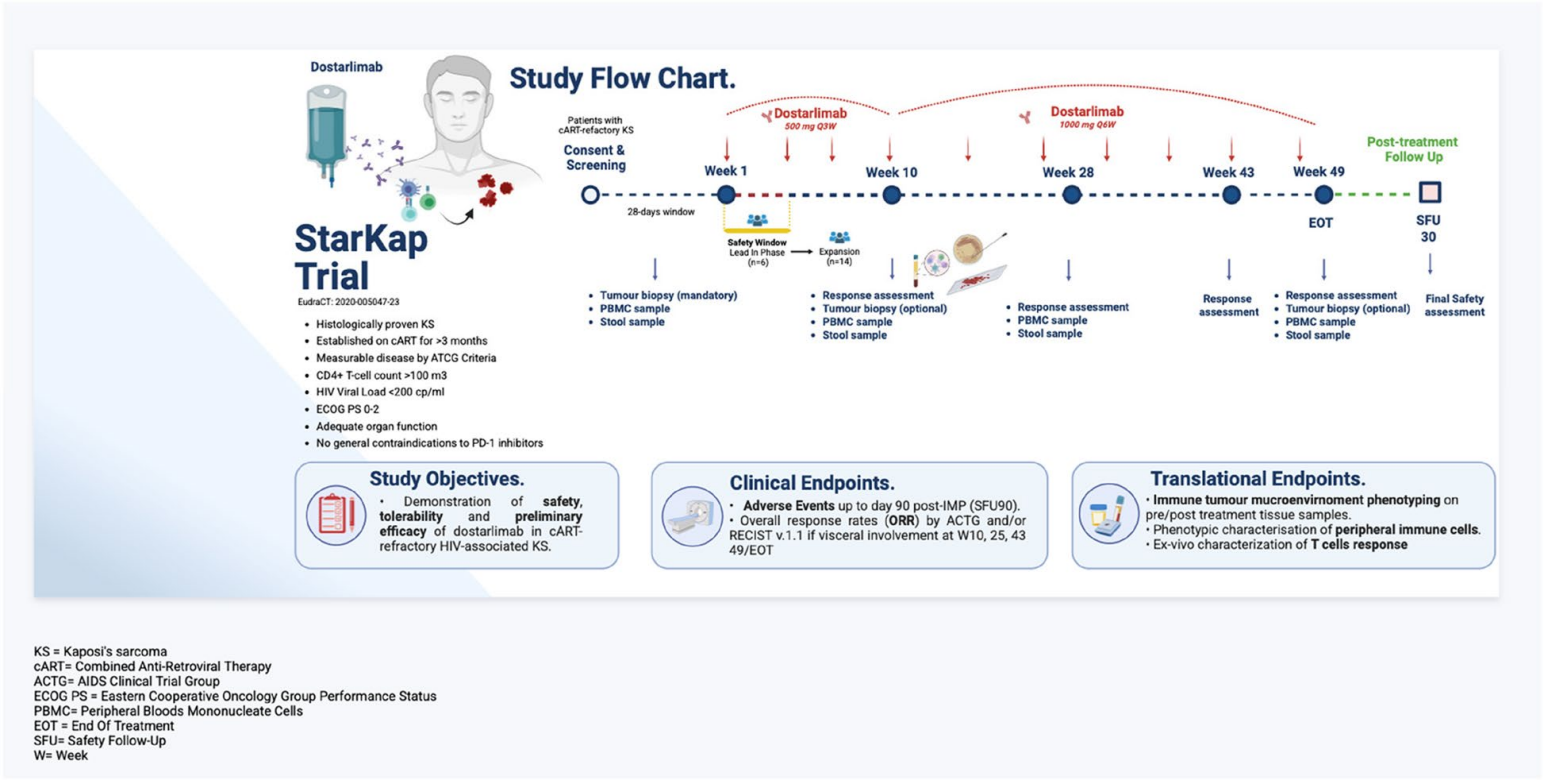
Trial flowchart

The trial scheme is summarised in Fig. 1.

### Inclusion and exclusion criteria

The trial will enrol patients affected by HIV-associated KS, requiring systemic therapy for KS. Patients will be required to be established on cART for HIV by at least 3 months, with CD4 count ≥ 100 cells/µL, and HIV-RNA < 200 copies/ml at screening. Patients are eligible independently from the previous exposure to chemotherapy for KS. Patients already exposed to immune-checkpoint inhibitors will be excluded.

The main inclusion and exclusion criteria are summarised in Table 2.

**Table 2:** Summary of inclusion and exclusion criteria

**Inclusion Criteria**
1.Histologically proven diagnosis of Kaposi’s Sarcoma (KS) fulfilling the clinical criteria for cART-refractory disease: defined as KS with clinical progression or stable disease after at least 3 continuous months of combined anti-retroviral, with virological and immunological response (defined at 4) 2.Available pre-treatment biopsy 3.Established on cART for at least 3 months without adverse events (AEs) associated with cART > grade 1 by National Cancer Institute (NCI) Common Terminology Criteria for Adverse Events (CTCAE) criteria version (v.) 5.0. Note: modifications of cART during screening are allowed provided patients meet all other eligibility criteria and are on effective new regimen for at least 2 weeks 4.HIV viral load < 200 viral copies/ml and CD4 + T-cell count ≥ 100 cells/µL at screening 5.Measurable disease by modified AIDS Clinical Trial Group (ATCG) Kaposi sarcoma response criteria 6.Eastern Cooperative Oncology Group (ECOG) performance status of ≤ 2 7.Be of ≥ 18 years of age 8.Adequate haematological and organ function, defined as follows: -Absolute neutrophil count > 1.5 × 10 ꝰ/L -Platelet count > 100.000/mcL, -Haemoglobin > 90 g/L, -Total bilirubin ≤ 1.5 × upper limit of normal (ULN) or ≤ 2xULN for patients with Gilbert’s syndrome or on HIV protease inhibitors, -Aspartate aminotransferase (AST)/alanine aminotransferase (ALT) ≤ 2.5 × ULN (up to 5 × ULN if liver metastases are present), -Serum creatinine ≤ 2.5 × ULN or creatinine clearance (CrCl) > 60 ml/min in subjects with serum creatinine ≤ 1.5 × ULN. Calculation of CrCl should follow institutional standards -International normalized ratio (INR) or prothrombin time (PT) ≤ 1.5 × ULN unless patient is receiving anticoagulant therapy as long as PT or partial thromboplastin (PTT) is within therapeutic range of intended use of anticoagulants. Activated partial thromboplastin time (aPTT) ≤ 1.5 × ULN unless patient is receiving anticoagulant therapy as long as PT or PTT is within therapeutic range of intended use of anticoagulants 9.Female participants must have a negative serum pregnancy test within 72 h prior to taking study treatment if of childbearing potential and agree use a highly effective method of contraception from screening through 120 days after the last dose of study treatment or is of non-childbearing potential 10.Participant must agree to not breastfeed during the study or for 90 days after the last dose of study treatment 11.Male participant agrees to use a highly effective method of contraception (see Sect. 4.4 for a list of acceptable birth control methods) starting with the first dose of study treatment through 120 days after the last dose of study treatment. Note: Abstinence is acceptable if this is the established and preferred contraception for the patient 12.Participant receiving corticosteroids may continue as long as their dose is stable for least 4 weeks prior to initiating protocol therapy 13.Participant must be able to understand the study procedures and agree to participate in the study by providing written informed consent
**Exclusion criteria**
1.Receipt of anti-cancer therapy (chemotherapy, radiation therapy, immunotherapy or biologic therapy) including investigational therapy ≤ 4 weeks, or within a time interval less than at least 5 half-lives of the investigational agent, whichever is shorter, prior initiating protocol therapy. There should be no evidence of treatment-related adverse events > grade 1 CTCAE v.5 2.Major surgery within 3 weeks prior to initiating protocol therapy. Study participant must have recovered from any adverse events relating to surgery 3.Known active tuberculosis (TB) in the first 6 weeks of treatment 4.Known active Immune Reconstitution Inflammatory Syndrome (IRIS) related to opportunistic pathogens. Note: Patients with previous history of IRIS that has fully resolved at the time of screening are eligible 5.Known history of active uncontrolled hepatitis C virus (HCV) infection, defined as detectable plasma HCV RNA. Note: patients with positive HCV serology but negative HCV RNA or those successfully treated for HCV are eligible 6.Known history of active uncontrolled hepatitis B virus (HBV) infection, defined as detectable plasma HBV DNA in absence of therapy. Note: patients with HBV serology indicating immunization (i.e. positive hepatitis B surface antibody, HBsAb and negative core antibody HBcAb), patients with fully resolved acute HBV infection and those with chronic HBV infection adequately treated with antiviral therapy are eligible 7.Active auto immune disease that has required systemic treatment in the past 2 years (i.e. with use of disease modifying agents, corticosteroids or immunosuppressive drugs). Replacement therapy (eg. thyroxine, insulin, or physiologic corticosteroid replacement therapy for adrenal or pituitary insufficiency, etc.) is not considered a form of systemic treatment 8.History of idiopathic pulmonary fibrosis, organizing pneumonia, drug-induced pneumonitis, or idiopathic pneumonitis, or evidence of active pneumonitis on screening chest computed tomography scan 9.Prior therapy with anti-PD-1/PD-L1 and/or anti-CTLA-4 therapy either alone or in combination with other agents 10.Receipt of live vaccines within 30 days before the first dose of trial treatment and while participating in the trial; examples of live vaccines include, but are not limited to, the following: measles, mumps, rubella, chicken pox, yellow fever, seasonal flu, H1 N1 flu, rabies, bacillus Calmette-Guerin (BCG), and typhoid vaccine 11.Known hypersensitivity to dostarlimab components or excipients 12.Known psychiatric or substance abuse disorders that would interfere with cooperation with the requirements of the trial 13.Known additional malignancy that is progressing or requires active treatment. Exceptions include basal cell carcinoma of the skin or squamous cell carcinoma of the skin that has undergone potentially curative therapy or in situ cervical cancer 14.Participant has leptomeningeal disease, carcinomatous meningitis, symptomatic brain metastases, or radiologic signs of CNS hemorrhage 15.Systemic steroid therapy or any other form of immunosuppressive therapy that cannot be discontinued within 7 days prior to initiating protocol therapy

## Objectives and endpoints

The main objective of the StarKap trial is to test the safety and the tolerability of dostarlimab in patients affected by cART-refractory KS. Efficacy is the secondary objective of the study. Along with the clinical objectives, we have designed and planned a set of translational objectives to identify the immunological correlates of response to dostarlimab in cART-refractory Kaposi’s sarcoma. Table 3 summarises the objectives and endpoints of the study.

**Table 3:** Trial Objectives and endpoints

Objective	Endpoint
**Primary**	
To determine the safety and tolerability of dostarlimab in cART-refractory HIV associated Kaposi Sarcoma	Incidence of adverse events measured by NCI CTC criteria v.5.0 up to 90 days after treatment cessation
**Secondary**	
A) To characterize the efficacy of dostarlimab in cART-refractory HIV associated Kaposi Sarcoma	1. Objective Response rate (ORR) evaluated at week 10 and week 31 (± 7 days) and end of treatment by modified AIDS Clinical Trial Group (ACTG) Kaposi sarcoma (KS) response criteria. For patients with visceral involvement Response Evaluation Criteria In Solid Tumours (RECIST) v1.1 will be used to complement modified ACTG response criteria 2. Progression free survival (PFS) from screening 3. Time to progression from the first evidence of documented objective response to dostarlimab
**Exploratory**	
A) To identify immunological correlates of response to dostarlimab in cART-refractory HIV associated Kaposi Sarcoma B) To define the impact of microbiome composition on response to dostarlimab C) To assess quality of life in the study population	1. Assessment of the phenotype of intra-tumour and peripheral immune cells at different timepoints and to correlated with ORR 2. Assessment of the transcriptional profile of intra-tumoral immune cells at different timepoints and to correlate with ORR 3. The dynamic changes of anti HHV-8 T cells response during treatment 4. The differences in the baseline microbiome composition according to ORR 5. To evaluate the changes in quality of life parameters during treatment

### Translational investigational plan

The hypothesis that guides the translational biomarker plan associated with StarKap trial is centred on the role of effector tumour infiltrating T-cell as a mechanism underscoring disease-modulating activity from immunotherapy in KS. We hypothesise dostarlimab to elicit anti-tumoral response by reinvigorating the T-cells effector function within the tumour microenvironment. To this end, tumour, bloods and stools samples will be longitudinally collected.

- A) Tumour samples
  Tumour biopsy will be mandatory at screening. Optional tumour samples will be collected at cycle and at the end of treatment to characterise the phenotype and the transcriptional profile of the intra-tumoral immune infiltrate.
  The phenotype of the intra-tumoral immune system will be assessed by performing imaging mass cytometry using a pre-defined panel of 30 markers specific for both the innate and adaptive immune system. The relative expression of the markers of interest will be evaluated pairwise in pre-on treatment samples and analysed in relationship with response to treatment.
  Transcriptomic profiling will be conducted in parallel with imaging mass cytometry experiments on tumour samples. Patients with a partial response will be categorised as “responders,” while those with stable or progressive disease will be classified as “non-responders.” Pathway enrichment analysis and immune deconvolution analysis will be conducted comparatively between the two groups. The same comparisons will be performed between pre-treatment and on-treatment samples.
  Available evidence suggest that HHV-8 specific T cells response is pivotal to prevent KS development. To verify whether immunotherapy elicits anti-tumour response by inducing clonal T-cells expansion, we will perform targeted sequencing of the rearranged VDJ segments on the CDR3 region of the TCRBeta chain on DNA extracted from FFPE pre-treatment and on-treatment tumour samples.
- B) Bloods samples
  Extra bloods samples will be collected at screening, cycle 4, cycle 8 and end of treatment.
  We will characterize baseline immune phenotype of PBMC and their changes during treatment using multi- parameter flow-cytometry. Mean and median values of the diverse immune cell populations detected by flow-cytometry will be measured and compared across timepoints, and between responders and non-responders.
  To investigate whether response to immunotherapy is associated with specific immune-response, we will perform targeted sequencing of the rearranged VDJ segments on the CDR3 region of the TCRBeta chain on DNA extracted T-cell receptor beta- chain (TCRB) repertoire in peripheral blood lymphocytes to assess for dynamic changes in clonality of the T- cell response.
  To assess whether response to anti PD-1 (defined as reported in the clinical objectives) is associated with a reduction in HHV-8 viral replication and increased immune response against HHV-8 we will evaluate the changes in HHV-8 DNA level by real-time PCR from whole blood samples at baseline, cycles 4, cycle 8 and at the EOT.
  To confirm determine if response to KS is associated with increase T-cells response against HHV-8, we will perform ex-vivo T-cells stimulation with a pool of overlapping peptides spanning selected HHV-8 reading frames (ORF) and assess the immune response using interferon gamma assay.
- C) Stools samples
  The functional interaction between commensal bacteria and host immune system has been widely recognized in humans and animal models [18]. HIV infection has been reported to alter the composition of the intra-mucosal T cells with consequent alteration of the intestinal barrier permeability and increased translocation of bacterial products into the blood stream [19]. Furthermore, the presence of alterations within the intestinal immune environment, has been reported to alter the composition of the gut microbiome in people living with HIV, and this has been associated with persistent chronic inflammation and peripheral T cells activation [20]. At the same time, the lack of microbiome diversity and the enrichment of specific taxa of commensal bacteria including Bifidobacteria, Akkermansia and Ruminococcaceae has been associated with reduced responsiveness to immunotherapy in patients with advanced malignancies through a direct effect of gut microbiome products on peripheral and intra-tumoral immune cells [21].
  To verify if the composition of the stool microbiome correlates with intra-tumoral and peripheral immune cells phenotype, and with response to dostarlimab, we will longitudinally characterize gut microbiome by sequencing hypervariable V1-V2 region of 16S rRNA gene from stool DNA collected at baseline.

### Statistical analysis

With safety being elected as primary clinical endpoint for the study, no power calculation for hypothesis testing was required to formally power the study. Justification for a sample size of 20 subject was based on previous experience in the use of pembrolizumab in HIV-associated cancers [9, 22].

An interim analysis on adverse events will be conducted after the 6 th subject clears the 21-days safety window. Pending review of safety data by the trial steering committee in the first 6 subjects, in the absence of significant toxicity, the study will continue to the recruitment of the remaining 14 subjects without pause.

The final efficacy analysis on the whole will be conducted at the end of the study after the last patient has completed the treatment.

The safety population will include all subjects receiving at least one dose of dostarlimab. All participants who receive at least one dose of dostarlimab and undergo efficacy evaluation will be included in the efficacy population. No patients will be replaced as this would introduce bias; drop-out is likely to be informative in this population.

The primary outcome will be presented using descriptive measures of safety such as frequencies and percentages of adverse events grouped as categorical variables and means and standard deviations for normally distributed continuous variables.

Efficacy endpoints will be estimated descriptively by documenting ORR and progression free survival rates (PFSR) at 12-weekly timepoints. Progression free survival will be estimated using the Kaplan–Meier method from screening to progression or death. The PFSR estimates the proportion of participants who do not progress and are alive at 12 weeks timepoint.

## Conclusions

HIV-associated KS, despite the widespread availability of cART to control HIV, remains the most common malignancies in PLWH, and few studies have been conducted in this setting.

We have designed the StarKap trial to define the role of immune-checkpoint inhibitors in PLWH and KS requiring systemic therapy. Whilst non-randomised and drawing on a sample size of only 20 patients, this trial will help delineating characteristics of response including ORR by standard ACTG KS criteria, and RECIST and describe overall depth and durability of response.

Furthermore, the translational plan of the trial will provide insights to understand the activity of immunotherapy on KS and the associated microenvironment. Systematic interrogation of a rich biorepository of tumour and peripheral samples will allow identification of pathways potentially involved in adaptive resistance to immunotherapy. This has the potential to facilitate the identification of novel targets for further therapeutic development.

## Data Availability

No datasets were generated or analysed during the current study.
